# Lymphoepithelioma-like carcinoma of the urinary bladder: a case report and review of the literature

**DOI:** 10.1186/1756-0500-7-779

**Published:** 2014-11-04

**Authors:** Tateki Yoshino, Shinya Ohara, Hiroyuki Moriyama

**Affiliations:** Department of Urology, JA Onomichi General Hospital, Hiroshima, 1-10-23 Hirahara, Onomichi, Hiroshima, 722-0018 Japan; Department of Urology, Integrated Health Sciences, Institute of Biochemical and Health Sciences, Hiroshima University, Hiroshima, Japan

**Keywords:** Lymphoepithelioma-like carcinoma, Urinary bladder, Bladder-preservation treatment

## Abstract

**Background:**

Lymphoepithelioma-like carcinoma is an undifferentiated carcinoma with histological features similar to undifferentiated, non-keratinizing carcinoma of the nasopharynx. Lymphoepithelioma-like carcinoma of the urinary bladder is uncommon with a reported incidence of 0.3%– 1.3% of all bladder cancer. We report a Japanese case of predominant lymphoepithelioma-like carcinoma of the urinary bladder and review all of the English literature after performing a pooled analysis of the cases including the present one.

**Case presentation:**

An 83-year-old Japanese man was introduced to our department with the chief complaint of macroscopic hematuria. Cystoscopy demonstrated a thumb tip-sized bladder tumor at the trigone. The patient underwent a transurethral resection of the bladder tumor. The pathological examination showed predominant lymphoepithelioma-like carcinoma of the urinary bladder with urothelial carcinoma. The patient was diagnosed with muscle invasive lymphoepithelioma-like carcinoma of the urinary bladder and was treated with concurrent chemoradiotherapy. The patient is under observation with regular clinical follow-up and remains well after 12 months, with no evidence of disease recurrence. The reports of 93 patients including the present one of lymphoepithelioma-like carcinoma of the urinary bladder from the English literature were collected between 1991 and 2014. Patients were evaluated for clinicopathological findings. Outcome resulted as follows: 59 patients (67%) did not show evidence of disease, 14 (17%) died of disease, 5 (6%) was alive with metastases, and 9 (10%) died for causes unrelated to the primary disease. Cause-specific survival rate resulted 83%. The overall patients were divided into three groups (pure, predominant and focal) according to the lymphoepithelioma-like carcinoma of the urinary bladder classification of Amin *et al*.

**Conclusions:**

Because lymphoepithelioma-like carcinoma of the urinary bladder is more sensitive to both chemotherapy and radiotherapy than conventional urothelial carcinoma, radical cystectomy may not be necessary for all patients with muscle invasive lymphoepithelioma-like carcinoma of the urinary bladder. Therefore, pathological information may be useful in selecting patients suitable for bladder-preservation treatment. On the other hand, the apparently more aggressive nature of focal lymphoepithelioma-like carcinoma of the urinary bladder suggests that these patients are probably best managed with radical cystectomy and adjuvant treatment.

## Background

Undifferentiated, non-keratinizing carcinoma with prominent lymphocytic infiltrate arising outside of the nasopharynx is called lymphoepithelioma-like carcinoma (LELC). Although LELC occurs in organs, such as salivary glands, uterine cervix, thymus, lung, skin, stomach, and breast, its occurrence in the urinary system is very rare. LELC of the urinary bladder (LELCB) has a reported incidence of 0.3%– 1.3% of all bladder cancer [1–3]. Contrary to nasopharynx cases, no relationship with Epstein-Barr virus has been reported. According to the lymphoepithelioma component, these tumors were classified as pure (100%), predominant (≥50%), and focal (<50%), advocated by Amin *et al*. [4].

Because of the small number of cases reported in the literature, there are no clear guidelines for the treatment of LELCB. Several reports suggest that, unlike conventional urothelial carcinoma and focal LELCB, the pure and predominant LELCB, irrespective of stage, are sensitive to chemotherapy and radiotherapy. This observation raises the potential of salvaging bladder function in patients with these subtypes [1, 2, 4]. This advantage is lost when the urothelial elements predominate.

## Case presentation

An 83-year-old Japanese man was introduced to our department with the chief complaint of macroscopic hematuria. There was a past medical history of hepatitis B and hepatocellular carcinoma treated with radiofrequency ablation, but no history of urological problems. Physical examination and vital signs were unremarkable.

Urinalysis showed combined hematuria and pyuria, but urine culture was sterile. Urine cytology indicated atypical cells. Abnormal hematological findings include leukocytosis (WBC count: 15,700/μl) and eosinophilia (12%). The serum IgE level was 9.8 IU/ml (normal <173 IU/ml).

**Figure 1:**
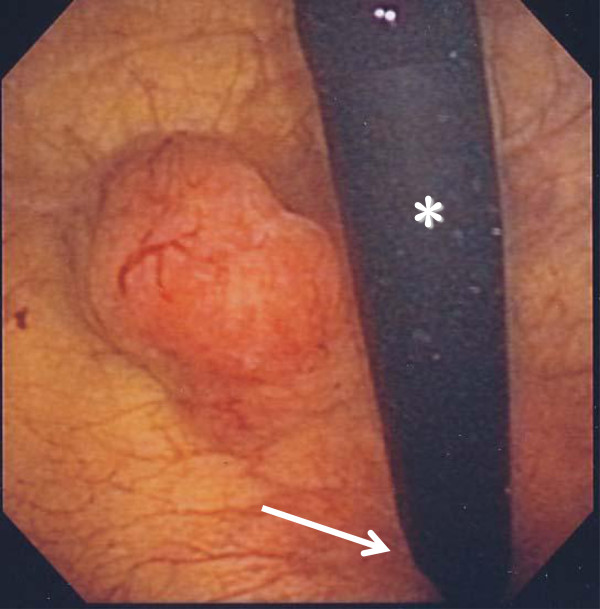
**Cystoscopy revealed a thumb tip-sized bladder tumor at the trigone.** The tumor was solid and broad-based. asterisk, flexible cystoscope; arrow, neck of bladder

**Figure 2:**
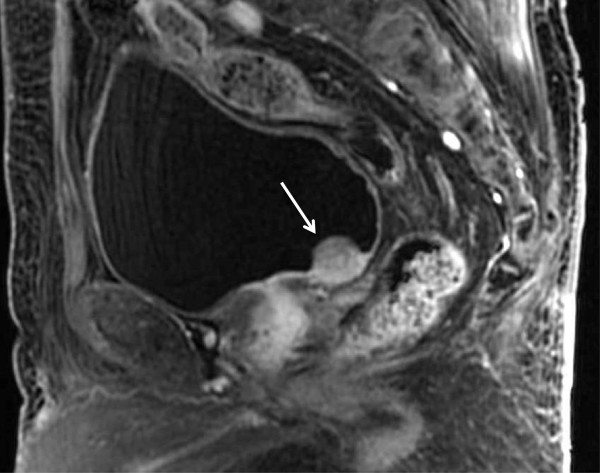
**Contrast-enhanced magnetic resonance imaging (sagittal image) showed a bladder tumor (arrow) at the trigone, 20 × 17 × 15 mm in size, with muscle invasion.**

**Figure 3:**
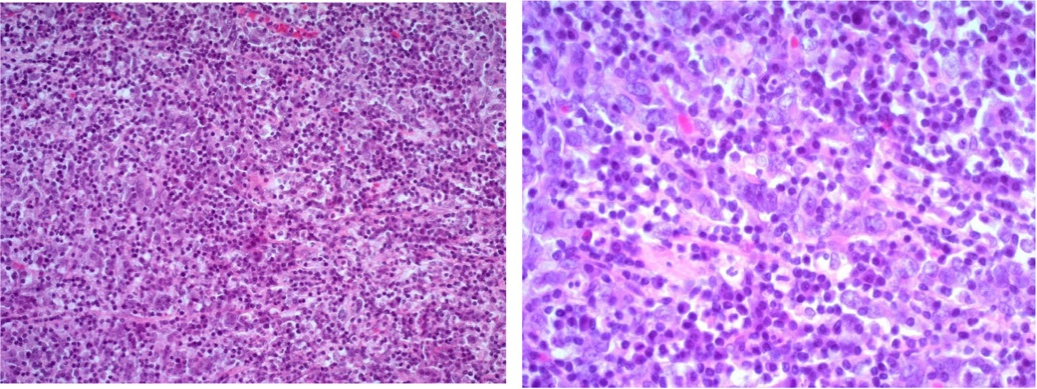
**Microscopic findings (left, ×100; right, ×400).** Lymphoepithelioma-like carcinoma, syncytial pattern with prominent lymphocytic infiltrate and lymphoepithelioma-like carcinoma with urothelial carcinoma.

**Figure 4:**
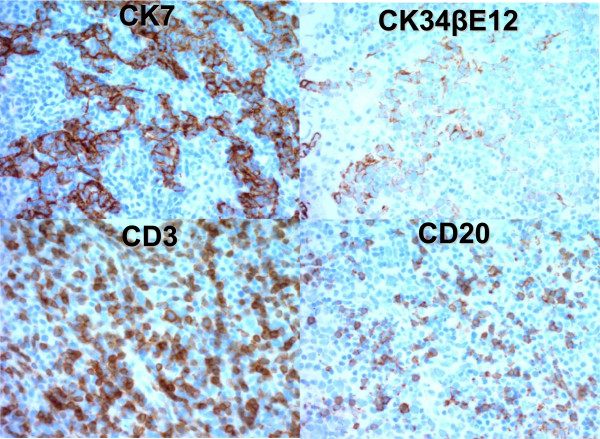
**Immunohistochemical findings of CK7, CK34βE12, CD3, and CD20.** The epithelial component revealed overexpression of CK7. Focal staining for CK34βE12 was detected. Positive staining with antibodies against CD3 and CD20 showed abundant T-lymphocytes and less B-lymphocytes, respectively.

Cystoscopy demonstrated a thumb tip-sized bladder tumor at the trigone (Figure 1) and papillary mucosa at the left lateral wall. Contrast-enhanced magnetic resonance imaging (MRI) showed a 20 × 17 × 15 mm bladder tumor at the trigone and muscle invasion in the bladder (Figure 2). Computed tomography (CT) revealed no distant metastases. The patient underwent transurethral resection of the bladder tumor (TURBT). Histopathological examination revealed that 90% of the volume of the tumor was LELC marked by syncytial growth pattern (Figure 3), heavy lymphocytic infiltrate, necrosis, brisk mitosis and muscle invasion. The epithelial cells expressed CK7 and CK34βE12, and the lymphocytes CD3 and CD20 (Figure 4). The rest of the tumor was composed of non-invasive urothelial carcinoma.

The patient received concurrent chemoradiotherapy. The chemotherapy regimen included gemcitabine administered at 1000 mg/m^2^ on Days 1 and 8 and cisplatin 70 mg/m^2^ on Day 1 of a 21-day cycle. Radiotherapy, 60 Gy in total, was started on Day 1, concurrently with chemotherapy.

Ten months after his initial visit to our department, the patient underwent second TURBT for evaluation of pathological effect. No viable cancer cells were detected in the bladder specimens, including the muscle layer. At the time of this writing, the patient is under observation with regular clinical follow-up and remains well after 12 months, with no evidence of disease recurrence.

## Materials and methods

On the basis of the review from 1991 to 2002 by Porcaro AB *et al*. [5], we added subsequent LELCB reports to the review and summarized. Conduct of this study conformed to the Declaration of Helsinki, and was approved by our institutional ethical committee (Institutional Review Board of Onomichi General hospital). The reports of 93 patients including the present one of LELCB from the English literature were collected from 1991 to 2014, as shown in Table 1 [1–23]. Patients were evaluated for age, sex, primary and adjuvant treatments, pathological stage, follow-up and outcome, and cause-specific survival. Patients underwent cystoscopy and transurethral resection of the tumor for both biopsy or complete removal of the mass. Tumor specimens were evaluated for local pathological stage (available in 92 cases), histological subtype according to the classification of Amin et al. for LELCB. LELCB was classified as pure when 100% of the tumor showed lymphoepithelioma-like carcinoma pattern, and predominant or focal when associated with usual urothelial carcinoma, adenocarcinoma, or squamous cell carcinoma. Mixed subtype in Table 2 was used synonymously with predominant or focal [6]. Primary treatments performed included TURBT, partial cystectomy, and radical cystectomy. Adjuvant treatments, when performed, included several courses of systemic multiagent chemotherapy with MVAC (methotrexate, vinblastine, doxorubicin, and cisplatin) or GC (gemcitabine, cisplatin), as well as intravesical chemotherapy with doxorubicin or bacillus Calmette-Guerin, and radiotherapy.

**Table 1:** **Reports of lymphoepithelioma-like carcinoma of the urinay bladder from the English literature including the present case**

Case no.	Reference	Year	Sex	Age	Chief complaint	Type	Stage	Treatment	Follow-up	Outcome
1	Zukerberg	1991	M	76	NA	PU	NA	TURBT, RT	NA	NA
2	Young	1993	M	81	NA	PU	T3	PC, CH	41	DOD
3	Dinney	1993	M	52	GH	PU	T2	TURBT, CH	72	NED
4	Dinney	1993	M	68	GH	PU	T2	TURBT, CH	60	NED
5	Dinney	1993	M	63	NA	PU	T2	TURBT, CH	11	NED
6	Amin	1994	M	52	GH	PU	T2	TURBT, CH	72	NED
7	Amin	1994	M	68	GH	PU	T2	TURBT, CH	60	NED
8	Amin	1994	M	63	GH	PU	T2	TURBT, CH	11	NED
9	Amin	1994	F	71	GH	PR	T2	TURBT, CH	9	NED
10	Amin	1994	F	67	GH	PR	T3	PC, RT	36	NED
11	Amin	1994	M	55	GH	PR	T2	RC	10	NED
12	Amin	1994	M	71	GH	PR	T2	RC	6	NED
13	Amin	1994	F	79	GH	PR	T3	RC	2	NED
14	Amin	1994	M	78	GH	F	T2	RC, CH	84	DOD
15	Amin	1994	M	66	GH	F	T3	RC	6	DOD
16	Amin	1994	M	68	GH	F	T1	RC	0	DWD
17	Bianchini	1996	M	72	NA	PU	T3	RC, CH	29	NED
18	Holmäng	1998	F	61	GH	PU	T2	TURBT, RT	216	DWD
19	Holmäng	1998	M	78	GH	PU	T1	TURBT, RT	13	DWD
20	Holmäng	1998	M	65	GH	PU	T2	TURBT	24	NED
21	Holmäng	1998	F	71	GH	PR	T3	TURBT, RT	21	DWD
22	Holmäng	1998	F	60	GH	PR	T3	RC, RT	104	NED
23	Holmäng	1998	F	65	GH	PR	T3	PC	76	NED
24	Holmäng	1998	F	84	GH	F	T1	TURBT	66	DOD
25	Holmäng	1998	M	72	GH	F	T3	RC, RT	68	DOD
26	Holmäng	1998	M	71	GH	F	T3	TURBT, RT	9	DOD
27	Constantinides	2001	M	NA	GH, Urgency	PU	T2	TURBT, CH	34	NED
28	Constantinides	2001	M	NA	GH, Urgency	PU	T1	TURBT, IV-CH	28	NED
29	Constantinides	2001	M	NA	GH, Urgency	PR	T2	RC	32	NED
30	Lopez-B	2001	F	69	NA	PU	T2	TURBT, CH	21	NED
31	Lopez-B	2001	M	72	NA	PU	T3	RC	32	NED
32	Lopez-B	2001	M	81	NA	PU	T2	TURBT, CH	47	NED
33	Lopez-B	2001	M	69	NA	PR	T2	TURBT	22	NED
34	Lopez-B	2001	F	67	NA	PR	T2	RC	22	NED
35	Lopez-B	2001	M	73	NA	PR	T3	RC	37	NED
36	Lopez-B	2001	M	82	NA	PR	T2	TURBT	49	NED
37	Lopez-B	2001	M	74	NA	PR	T2	TURBT	25	AWM
38	Lopez-B	2001	F	81	NA	PR	T2	TURBT	44	DOD
39	Lopez-B	2001	M	78	NA	F	T2	TURBT	3	DOD
40	Lopez-B	2001	F	58	NA	F	T2	RC	19	DOD
41	Lopez-B	2001	M	71	NA	F	T2	TURBT	30	DOD
42	Lopez-B	2001	M	69	NA	F	T3	RC	18	DOD
43	Ward	2002	M	61	GH	PR	T2	TURBT	NA	NA
44	Porcaro	2003	M	72	NA	PR	T2	TURBT	17	NED
45	Chen	2003	M	73	GH	PU	T3	RC, CH	26	NED
46	Chen	2003	M	63	GH	PU	T1	TURBT, IV-CH	26	NED
47	Izuquierdo	2004	M	77	GH	PU	T2	TURBT, RT	39	NED
48	Izuquierdo	2004	F	82	GH	PR	T2	TURBT	36	NED
49	Izuquierdo	2004	M	74	GH	F	T2	TURBT, RT	54	NED
50	Guresci	2005	M	90	GH	PR	T3	TURBT	NA	NA
51	Abascal	2005	M	54	GH	NA	T3	NA	NA	NA
52	Yaqoob	2005	F	55	GH, Miction pain	NA	T2	TURBT	NA	NA
53	Mayer	2007	M	57	GH	PU	T1	RC	36	NED
54	Tamas	2007	NA	NA	NA	PU	T3	PC	23	AWM
55	Tamas	2007	NA	NA	NA	PU	T2	RC	40	NED
56	Tamas	2007	NA	NA	NA	PU	T4	RC, RT	36	NED
57	Tamas	2007	NA	NA	NA	PU	T3	RC	42	NED
58	Tamas	2007	NA	NA	NA	PU	T2	RC	46	NED
59	Tamas	2007	NA	NA	NA	PU	T4	RC, CH	29	NED
60	Tamas	2007	NA	NA	NA	PU	T3	RC	23	NED
61	Tamas	2007	NA	NA	NA	PU	T2	RC	3	DWD
62	Tamas	2007	NA	NA	NA	PU	T2	TURBT, CH, RT	17	NED
63	Tamas	2007	NA	NA	NA	PU	T2	TURBT, Thermal, CH	4	NED
64	Tamas	2007	NA	NA	NA	PU	T1	TURBT, RT	6	NED
65	Tamas	2007	NA	NA	NA	PU	T3	RC	4	NED
66	Tamas	2007	NA	NA	NA	PU	T2	TURBT, RT, CH	65	DWD
67	Tamas	2007	NA	NA	NA	PU	T1	TURBT, IV-CH	38	NED
68	Tamas	2007	NA	NA	NA	PU	T1	TURBT	48	DWD
69	Tamas	2007	NA	NA	NA	PU	T2	RC	2	NED
70	Tamas	2007	NA	NA	NA	Mixed	T3	RC	55	NED
71	Tamas	2007	NA	NA	NA	Mixed	T2	TURBT, RT, CH	24	NED
72	Tamas	2007	NA	NA	NA	Mixed	T2	PC	10	NED
73	Tamas	2007	NA	NA	NA	Mixed	T2	TURBT	4	AWM
74	Tamas	2007	NA	NA	NA	Mixed	T1	TURBT, CH	6	NED
75	Tamas	2007	NA	NA	NA	Mixed	T1	TURBT	42	DWD
76	Tamas	2007	NA	NA	NA	Mixed	T1	RC	9	NED
77	Tamas	2007	NA	NA	NA	Mixed	T2	TURBT, CH	2	NED
78	Tamas	2007	NA	NA	NA	Mixed	T2	TURBT, RT, CH	8	AWM
79	Tamas	2007	NA	NA	NA	Mixed	T1	RC	18	DOD
80	Tamas	2007	NA	NA	NA	Mixed	T3	RC	5	AWM
81	Tamas	2007	NA	NA	NA	Mixed	T2	PC	2	NED
82	Singh	2009	M	69	GH	PU	T2	RC	12	NED
83	Trabelsi	2009	M	58	GH	NA	T2	RC	NA	NA
84	Yun	2010	F	78	GH	PR	T1	TURBT	8	NED
85	Kozyrakis	2011	M	72	GH	PR	T2	TURBT, RT	72	NED
86	Kozyrakis	2011	M	75	GH	PR	T3	RC, CH	26	NED
87	Kozyrakis	2011	F	80	GH	PR	T2	TURBT, RT	13	NED
88	Kozyrakis	2011	F	69	GH	PR	T3	RC, CH	34	DWD
89	Kozyrakis	2011	M	70	GH	F	T2	TURBT	14	DOD
90	Kozyrakis	2011	M	72	GH	F	T2	TURBT, RT, CH	27	DOD
91	Pantelides	2012	M	64	GH	PU	T2	TURBT, CH	6	NED
92	Mori	2013	M	70	GH	PR	T2	RC	10	NED
93	Present case	2014	M	83	GH	PR	T2	TURBT, CH, RT	12	NED

*NA* Not available, *GH* Gross hematuria, *PU* pure, *PR* Predominant, *F* Focal, *TURBT* Transurethral resection of the bladder tumor, *PC* Partial cystectomy, *RC* Radical cystectomy, *CH* Chemotherapy, *RT* Radiotherapy, *IV*-*CH* Intravesical chemotherapy, *NED* Not evidence of disease, *DOD* Died of disease, *DWD* Died without disease, *AWM* Alive with metastases.

**Table 2:** **Lymphoepithelioma-like carcinoma of the urinary bladder: clinical and pathological results in 93 cases**

Features		Patient population
Sex	(n = 65)	
Male		48 (74%)
Female		17 (26%)
Age	(n = 62)	
Mean		70.0
Range		52-90
Chief complaint	(n = 35)	
GH		31 (88%)
GH and Urgency		3 (9%)
GH and Miction pain		1 (3%)
Histology	(n = 90)	
Pure		39 (43%)
Predominant		26 (29%)
Focal		13 (15%)
Mixed		12 (13%)
Pathological stage (T)	(n = 92)	
T1		14 (15%)
T2		52 (56%)
T3		24 (27%)
T4		2 (2%)
Primary treatment	(n = 92)	
TURBT		51 (55%)
Partial cystectomy		6 (7%)
Radical cystectomy		35 (38%)
Adjuvant treatment	(n = 92)	
Systemic chemotherapy		21 (23%)
Radiotherapy		14 (15%)
Chemoradiotherapy		6 (7%)
Intravesical chemotherapy		3 (3%)
Not performed		48 (52%)
Follow-up	(n = 87)	
Mean		30.4 (months)
Range		0-216 (months)
Outcome	(n = 87)	
Not evidence of disease (NED)		59 (67%)
Died of disease (DOD)		14 (17%)
Died without disease (DWD)		9 (10%)
Alive with metastases (AWM)		5 (6%)
Cause-specific survival rate		83%

*GH* Gross hematuria, *TURBT* Transurethral resection of the bladder tumor.

The overall patient population was separated into 3 groups according to the LELCB classification of Amin *et al*. Each group was evaluated for the same clinical, pathological features as described previously for the overall population group.

## Results

As shown in Table 1, 93 patients with LELCB were reported from 1991 to 2014. Table 2 shows the features of the overall patient population which included 48 males and 17 females. Mean age was 70.0 years (range: 52–90). Gross hematuria (GH) was seen in all available patients with presenting symptoms. LELCB histological subtypes resulted pure in 39 cases (43%), predominant in 26 (29%), focal in 13 (15%), and mixed in 12 (13%). Patients with pT2 and pT3 together accounted for 83% of the patient population. Primary treatments included TURBT in 51 patients (55%), partial cystectomy in 6 (7%), and radical cystectomy in 35 (38%). Adjuvant treatment, which was not performed in 48 cases (52%), included systemic chemotherapy in 21 (23%), radiotherapy in 14 (15%), chemoradiotherapy in 6 (7%), and intravesical chemotherapy in 3 (3%). Outcome resulted as follows: 59 patients (67%) did not show evidence of disease, 14 (17%) died of disease, 5 (6%) was alive with metastases, and 9 (10%) died for causes unrelated to the primary disease. Cause-specific survival rate resulted 83% in the overall patient population.

Table 3 shows the results related to the three groups of patients with LELCB according to their histological classification: pure (group 1), predominant (group 2), and focal (group 3). Mean age resulted about 70 years in all groups. Pathological stage T2 and T3 together was detected in 76–97% of patients in each group. More than 90% of patients underwent TURBT or radical cystectomy as the primary treatment in all groups. Adjuvant treatment was as follows: Systemic chemotherapy was performed in 15 patients (38%) of the group 1, 3 (12%) of the group 2, and 1 (8%) of the group 3. Radiation was performed in 6 patients (16%) of the group 1, 5 (19%) of the group 2, and 3 (23%) of the group 3. Chemoradiation was done as adjuvant treatment in 2 patients (5%) of the group 1, 1 (4%) of the group 2, and 1 (8%) of the group 3. Intravesical chemotherapy was performed in 3 patients (8%) of the group 1. Adjuvant treatment was not performed in 13 patients (33%) of the group 1, 17 (65%) of the group 2, and 8 (61%) of the group 3. Patients with no evidence of disease were more than 80% of patients in the group 1 and 2, whereas 8% in the group 3. Patients who died of their disease resulted 84% of patients in the group 3, whereas less than 5% in the group 1 and 2. Though cause-specific survival rate resulted more than 90% in the group 1 and 2 (mean follow-up 35.2 and 30.1 months, respectively), it was 15% in the group 3 (mean follow-up 30.6 months).

**Table 3:** **Lymphoepithelioma-like carcinoma of the urinary bladder: histological subgroups according to the classification of Amin** ***et al*** **.**

Features	Group 1- Pure	Group 2- Predominant	Group 3- Focal
Number of patients	39 (49%)	26 (34%)	13 (17%)
Male	21	14	11
Female	2	12	2
NA	16	-	-
Age			
Mean	67.8	72.7	71.6
Range	52-81	55-90	58-84
Stage			
T1	7 (18%)	1 (3%)	2 (15%)
T2	21 (54%)	16 (62%)	7 (54%)
T3	8 (22%)	9 (35%)	4 (31%)
T4	2 (6%)	-	-
Primary treatment			
TURBT	23 (59%)	14 (54%)	7 (54%)
Partial cystectomy	2 (5%)	2 (8%)	-
Radical cystectomy	14 (36%)	10 (38%)	6 (46%)
Adjuvant treatment			
Systemic chemotherapy	15 (38%)	3 (12%)	1 (8%)
Radiotherapy	6 (16%)	5 (19%)	3 (23%)
Chemoradiotherapy	2 (5%)	1 (4%)	1 (8%)
Intravesical chemotherapy	3 (8%)	-	-
Not performed	13 (33%)	17 (65%)	8 (61%)
Follow-up			
Mean	35.2	30.1	30.6
Range	2-216	2-104	0-84
Outcome			
Not evidence of disease (NED)	31 (81%)	20 (84%)	1 (8%)
Died of disease (DOD)	1 (3%)	1 (4%)	11 (84%)
Died without disease (DWD)	5 (13%)	2 (8%)	1 (8%)
Alive with metastases (AWM)	1 (3%)	1 (4%)	-
Cause-specific survival rate	97%	95%	15%

*GH* Gross hematuria, *TURBT* Transurethral resection of the bladder tumor.

## Discussion

LELCB affects primarily the elderly with male predominance, 74% of cases reported in the literature (Table 2). With respect to chief complaint, GH was observed in all available patients with presenting symptoms. Most patients with LELCB have muscle invasive T2–3 disease, regardless of histological classification (pure, predominant, and focal). In fact, as shown in Tables 2 and 3, LELCB patients with T2–3 stage resulted 83% in overall population, 76% in group 1 (pure), 97% in group 2 (predominant), and 85% in group 3 (focal). Despite its infiltrative predisposition, the metastatic potential of LELC seems to be low [1, 6]. LELCB is diagnosed at less advanced stages and has a more favorable long-term prognosis than other types of undifferentiated invasive carcinoma of the bladder.

The histological features of LELCB include an inflammatory infiltrate and a dense lymphocytic infiltrate; furthermore, the syncytial arrangement of large neoplastic epithelial cells with prominent nuclei and nucleoli can be observed [24, 25]. Once LELCB is identified, a primary tumor in the nasopharynx should be excluded with CK7 stain. Nasopharyngeal carcinomas do not express CK7. Other differential diagnosis usually includes malignant lymphoma, undifferentiated urothelial carcinoma with prominent lymphoid infiltrate, chronic cystitis, and small cell carcinoma of the bladder [1, 2, 4, 7]. These can be excluded by relevant immunohistochemistry [1, 2, 4, 8, 26].

In Table 2, a previous pooled analysis showed that at a mean follow-up of 30.4 months, 67% of the overall population did not show any evidence of disease with a cause-specific survival rate of 83%. The cause-specific survival rate was more than 90% for both pure and predominant LELCB (mean follow-up of 35.2 and 30.1 months, respectively), whereas it was 15% (mean follow-up 30.6 months) for focal LELCB. The pure and predominant subtypes are more favorable than the focal subtype in prognosis, which may be related to the inflammatory infiltrate that results in a strong immune response against the atypical cells [4], early presentation of lower urinary tract symptoms [2], and to the chemoradiosensitivity of the neoplastic cells. This prognosis may be related to the host response, seen as a dense infiltrate composed predominantly of T-lymphocytes in the tumor [4], similar to medullary carcinoma of the breast and seminoma which also contain T-lymphocytes.

To date, owing to the scarcity of reported cases, there are no clear guidelines for the treatment of LELCB whose biological behavior differs from that of conventional urothelial carcinoma. Primary treatments for LELCB include both TURBT and partial or radical cystectomy. LELCB is sensitive to both cisplatin-based chemotherapy and radiotherapy, both of which have been used as adjuvant treatments after TURBT or radical bladder surgery [1, 2, 4]. Adjuvant combination chemotherapy includes three to five courses of methotrexate, vinblastine, doxorubicin, and cisplatin [1, 2, 4, 9, 26]. As shown in Table 3, the highest mortality was detected in the patients with focal LELCB who had local radical surgery, which was not followed by any adjuvant treatment in 61% of the patients. At about the same mean follow-up as focal subtype (group 3), 84% of the patients with predominant LELCB were alive and disease free although 65% of patients did not undergo any adjuvant treatment. These findings indicate that adjuvant treatment with chemotherapy and/or radiotherapy after local treatment of the primary tumor is advised for patients presenting with focal LELCB. Bladder-preservation therapy by performing both TURBT alone or combined with adjuvant chemotherapy and/or radiotherapy may be a reasonable option for patients with pure or predominant LELCB, whereas radical surgery with adjuvant treatment may be indicated for focal LELCB with muscle invasion. In addition, because the focal LELCB patients treated with even radical surgery alone had comparatively short survival (6, 18, and 19 months) in Table 1, we thought that adjuvant treatment was essential for procedure of focal LELCB. As shown in Tables 1 and 3, most patients (84%) with focal LELCB died of the primary tumor, whereas two patients of them, who underwent radical cystectomy and adjuvant treatment, had a relatively favorable outcome (64 and 84 months in survival). The apparently more aggressive nature of focal LELCB suggests that these patients are probably best managed with radical cystectomy and adjuvant treatment.

The present case was predominantly composed of LELCB accompanied by non-invasive urothelial carcinoma. On the basis of recommendations in the published literature, the patient underwent concurrent chemoradiotherapy after TURBT, leading to bladder-preservation. Twelve months after the initial visit, no evidence of disease recurrence was observed.

Previous reports and our experience suggest that radical cystectomy may not be necessary for all patients with muscle invasive LELCB and that radiotherapy and chemotherapy may be reliable treatment options. As Kozyrakis *et al*. [3] also said, pathological information might be useful in selecting patients suitable for bladder-preservation treatment. However, a large-scale study with long-term follow-up is needed to better understand the biological behavior of LELCB.

## Conclusion

Because LELCB is sensitive to both chemotherapy and radiotherapy, radical cystectomy may not be necessary for all patients with muscle invasive LELCB. Therefore, pathological information might be useful in selecting patients suitable for bladder-preservation treatment.

The apparently more aggressive nature of focal LELCB suggests that these patients are probably best managed with radical cystectomy and adjuvant treatment.

## Consent

Written informed consent was obtained from the patient for publication of this case report and any accompanying images. A copy of the written consent is available for review by the Editor-in-Chief of this journal.

## Abbreviations

LELC: Lymphoepithelioma-like carcinoma
LELCB: LELC of the urinary bladder
WBC: White blood cell
MRI: Magnetic resonance imaging
CT: Computed tomography
TURBT: Transurethral resection of the bladder tumor
GH: Gross hematuria
NA: Not available
PU: Pure
PR: Predominant
F: Focal
TURBT: Transurethral resection of the bladder tumor
PC: Partial cystectomy
RC: Radical cystectomy
CH: Chemotherapy
RT: Radiotherapy
IV-CH: Intravesical chemotherapy
NED: Not evidence of disease
DOD: Died of disease
DWD: Died without disease
AWM: Alive with metastases.
